# Evaluation of the Effect of Fungatol and Gamma-T-ol on the Emergence and Adult Parasitoid Survival of Mummies of Cotton Aphids Parasitized by *Aphidius colemani*

**DOI:** 10.3390/insects13010038

**Published:** 2021-12-29

**Authors:** Errol Hassan, Md Munir Mostafiz, Ellen Talairamo Iramu, Doug George, Kyeong-Yeoll Lee

**Affiliations:** 1 School of Agriculture and Food Sciences, The University of Queensland Gatton, Lawes, QLD 4343, Australia; douggeorge@cropconsult.com.au; 2 Division of Applied Biosciences, College of Agriculture and Life Sciences, Kyungpook National University, Daegu 41566, Korea; munirmostafiz12@gmail.com (M.M.M.); leeky@knu.ac.kr (K.-Y.L.); 3 Pacific Community (SPC)—Land Resources Division, Narere Campus, Suva, Fiji; elleni@spc.int; 4 Sustainable Agriculture Research Center, Kyungpook National University, Daegu 39061, Korea

**Keywords:** aphid parasitoid, parasitized cotton aphid mummies, survival of adult parasitoid, essential oils, sustainable agriculture

## Abstract

**Simple Summary:**

Biological control can be used as an alternative control measure to reduce pesticide resistance. Unfortunately, many biological control agents, such as natural enemies of pests, are susceptible to a broad spectrum of pesticides. This creates a potential problem when these two components are utilized together. Therefore, it is necessary to find alternatives that are not harmful to natural enemies but also have the potential to replace synthetic pesticides. Essential oils (EOs) are widely used in crop protection and organic agriculture. The EO formulations evaluated in this study are new botanical pesticides that play an important role in agriculture. EOs are available as an alternative to synthetic pesticides. Two blends (Fungatol and Gamma-T-ol) are mostly composed of Alpha Tops, and Gamma Tops were assessed for their effects on the aphid parasitoid *Aphidius colemani* in laboratory and glasshouse trials. According to the International Organization for Biological Control (IOBC) classification, they were found to be safe or only slightly toxic, making them potential candidates for introduction into an integrated pest control program for aphids.

**Abstract:**

Beneficial insects play a major role in controlling pest populations. In sustainable agricultural production systems, control methods compatible with integrated pest management (IPM) are preferred over broad-spectrum pesticides. EOs from aromatic plants may provide a new and safe alternative to synthetic chemicals. In this research, the efficacy of Fungatol, Gamma-T-ol, Fungatol plus neem, and Gamma-T-ol plus neem was evaluated against *Aphidius colemani* Viereck (Hymenoptera: Braconidae; Aphidiidae), the parasitoid of the cotton aphid, *Aphis gossypii* Glover (Hemiptera: Aphididae). Under laboratory and greenhouse conditions, five different concentrations of each formulation were applied to parasitized mummies and adult parasitoids. Results for parasitoid emergence from aphid mummies sprayed with different concentrations of Fungatol, Gamma-T-ol, Fungatol plus neem, and Gamma-T-ol plus neem in the laboratory and glasshouse showed that the formulations did not adversely affect adult emergence as rates above 60% were observed. For residual toxicity tests done by exposing adult parasitoids to a fresh, dry biopesticide film sprayed on glass plates, less than 20% mortality was observed after 48 h of exposure. Adult longevity tests revealed that the highest concentrations of some of the formulations evaluated were slightly toxic to *A. colemani*. According to the IOBC rating, our results indicated that most of the tested concentrations for each formulation were harmless to *A. colemani*. Based on the above results, it may be proposed that the formulations evaluated in this study are potential botanical pesticide candidates for incorporation into an IPM program.

## 1. Introduction

Aphid parasitoids (Hymenoptera: Aphidiinae) are important biological controls, effective in reducing populations of aphid species worldwide [1,2,3]. Aphidiinae members are obligatory, primary endoparasitoids of adult aphids [4,5]. The parasitoid deposits its egg into the aphid and, upon hatching, the larva feeds internally on the aphid, eventually resulting in its death. In Australia, twenty-one species have been reported [4]. An important species is *Aphidius colemani* Viereck (Hymenoptera: Braconidae; Aphidiidae), a pan-tropical species of parasitoid, widely distributed in Africa, Asia, Australia, South America, and Southern Europe, that parasitizes several species of Aphididae, including *Aphis gossypii* Glover and *Myzus persicae* Sulzer (Hemiptera: Aphididae) [6]. It is commercially available for the biological control of aphids [7,8].

Slippery kabis (*Abelmoschus manihot* L.) is one of the major vegetable crops extensively grown in the lowlands of Melanesian countries [9]. *A. Manihot* is one of the most significant traditional green vegetables, and its young leaves and shoots are consumed daily by the entire rural population in Melanesian countries [9]. It is a perennial shrub that belongs to the Hibisceae tribe of the Malvaceae family. It is reported as being attractive to many insect pests, classified into three major categories: leaf chewing, stem boring, and sap sucking [10]. Young shoots and succulent young leaves of the *A. Manihot* plant are highly susceptible to a number of chewing and sucking insect pests, including flea beetle (*Nisotra basselae* Bryant), tip borer (*Earias vitella* Fab.), and jassid (*Amrasca devastans* Dist.) [10,11]. In addition, *A. gossypii* populations have also been found infesting *A. manihot* plants in Australia [9].

In sustainable production systems, integrated pest management (IPM) approaches that employ both biological and chemical control agents against pests are preferred over pesticide-only approaches [12,13]. The use of non-selective chemical pesticides for pest control is detrimental to natural enemies, causing a reduction in their populations [14], resulting in pest resurgences [15,16]. Thus, it is important to use pesticides that have lethal and sublethal effects only on the target pest and are relatively harmless to natural enemies. The development of pesticides derived from natural sources that are less harmful to the environment than synthetic pesticides has been a focus of recent studies [17,18,19,20,21]. Botanical pesticides are considered to be good alternatives to synthetic pesticides because they are often less harmful to human health, the environment, and non-target beneficial insects [15,22,23,24,25,26,27,28,29]. Botanical pesticides are derived from plant species in many different families and are obtained either as plant extracts or essential oils (EOs) [30,31]. EOs and their components have insecticidal activity, making them viable IPM options [30].

One of the most important plant families in Australian flora is the Myrtaceae [32]. Several Myrtaceae spp., including *Melaleuca alternifolia*, *Backhousia citriodora*, and *Leptospermum petersonii*, are medicinal plants that are mostly found in Australia [33]. The insecticidal activities of EOs derived from these plants, particularly *M. alternifolia* (Tea tree oil, TTO), have been investigated against various pest insects [34,35,36,37]. The composition of TTO has almost 100 different chemicals, predominantly monoterpenes (terpinen-4-ol, terpinolene, p-cymene, α-pinene, γ-terpinene, 1,8-cineole), sesquiterpenes, and their respective alcohols (monoterpene, alcohol-terpineol) [35,38,39,40]. Fungatol, with Alpha Tops as the main constituent, comprises oxygenated terpenes, namely terpinen-4-ol, cineole, and alpha-terpineol, while Gamma-T-ol, with Gamma Tops, comprises non-oxygenated terpenes, viz. gamma-terpinene, terpinolene, and alpha-terpinene [9].

The insecticidal activity of Fungatol and Gamma-T-ol has been investigated previously against *A. gossypii*, *Helicoverpa armigera* Hübner (Lepidoptera: Noctuidae), *Tetranychus urticae* Koch (Acari: Tetranychidae), *Tuta absoluta* Meyrick (Lepidoptera: Gelechiidae), and *Phthorimaea operculella* Zeller (Lepidoptera: Gelechiidae) [9,41,42,43,44,45]. Furthermore, Iramu [9] showed improved efficacy against *A. gossypii* and *H. armigera* when Fungatol and Gamma-T-ol were combined with neem oil. However, the effects of these two blends, as well as the combination of these two blends with neem oil, on natural enemies have never been studied. As a result, it is critical to investigate the effects of these new botanical pesticides on natural enemies (e.g., *A. colemani*) before incorporating them into an IPM program. The goals of this study were to assess the effects of Fungatol and Gamma-T-ol, as well as the combination effects of these two blends with neem oil, on the emergence of *A. colemani* from treated aphid mummies in the laboratory and glasshouse, and to assess the pesticide residues’ effects on adult parasitoids’ survival and longevity in laboratory conditions.

## 2. Materials and Methods

### 2.1. Aphids and Parasitoids

The cotton aphid was obtained from field-grown, aphid-infested slippery kabis plants (Lockyer Valley, QLD, Australia). The aphid colony was reared on the slippery kabis plants in laboratory conditions. The colony was maintained without exposure to any insecticides. The *A. colemani* population for this study was initiated from cotton aphid mummies collected on *A. manihot* plants in the field (Lockyer Valley, QLD, Australia). The mummies were placed in 90 cm diameter Petri dishes to hatch; emerging female and male parasitoids were transferred to cages (45 cm × 45 cm × 45 cm) for mating and were fed 50% sucrose solution presented on cotton wicks placed in small glass tubes. Aphid-infested slippery kabis plants were placed in cages with parasitoids for parasitism. Parasitoid rearing was undertaken in the laboratory under controlled conditions with 20 ± 2 °C, 65–70% relative humidity, and a photoperiod of 16:8 h light:darkness [46]. Laboratory experiments with parasitoid mummies and adults were conducted under the same conditions as rearing.

### 2.2. Chemicals

Fungatol is made up of Alpha Tops (65%) and non-ionic surfactants (35%) and Gamma-T-ol of Gamma Tops (75%) and non-ionic surfactants (25%). The active ingredients of Alpha Tops (Fungatol) and Gamma Tops (Gamma-T-ol) employed in this study are listed in Table 1. Both formulations were solubilized in non-ionic surfactants. The formulations of these two blends used in this study were provided by BioAust Health Pty Ltd. in Australia. They were tested alone and in combination with neem oil. BioAust Health Pty Ltd., Australia, also provided the formulations of Fungatol plus neem, which contained 50% neem oil and 50% Fungatol, and Gamma-T-ol plus neem, which contained 50% neem oil and 50% Gamma-T-ol [9,38]. In both formulations, Tergitol^TM^ XD was employed as an emulsifier [9].

### 2.3. Effect of the Tested Formulations on A. colemani Emergence under Laboratory Conditions

To produce large numbers of mummies, 6–8-week-old slippery kabis plants infested with cotton aphid were transferred to cages. Mated *A. colemani* females were placed in each cage to parasitize aphids until mummies were observed. After mummification occurred, leaves with mummies were removed from the plants, and unhatched mummies were counted under a stereomicroscope.

Spraying mummies with different concentrations of formulations that could kill between 30 and 90% (LC30–LC90) of cotton aphids was used to evaluate the effect of formulations on the development of wasps in mummies (Table 2). Thirty to forty mummies were sprayed with ~0.5 mL (three times sprayed) of each specified concentration on slippery kabis leaf pieces using a hand-held glass spray bottle. Deionized water (~0.5 mL) was used as an untreated control. Each concentration was replicated four times. Therefore, a total of 120–160 mummies were tested for each treatment and the control.

Sprayed mummies were left to dry for thirty minutes and then transferred into Petri dishes (9 cm diameter), with the lid placed but not sealed, to provide ventilation for the developing wasp. The Petri dishes containing the aphid mummies were held in the laboratory under controlled conditions (Section 2.1). Adult emergence from sprayed mummies was recorded over 7 days and those that did not produce a wasp after this period were considered dead [6,27]. Adult emergence for each concentration was compared with the control to determine the effect of each formulation.

### 2.4. Effect of Tested Formulations on Parasitoid Emergence under Glasshouse Conditions

Glasshouse spraying of mummies was done to further test the effect of formulations on developing wasps within mummies under semi-controlled conditions, which might be more similar to the field than the laboratory environment. Procedures for mummy spraying in the glasshouse were adopted from Lowery [47] with modifications. The glasshouse conditions were 28 ± 2 °C with relative humidity of 70 ± 5%.

Mummy spraying in the glasshouse for each formulation was done on four groups of slippery kabis plants (4 plants/group), each plant bearing mummified aphids. Before spraying, the number of unhatched mummies on the leaves of each plant was counted with a hand lens and mummy numbers were recorded. A minimum of 30–50 mummies per plant was sprayed with three concentrations of Fungatol (0.25, 0.37, and 0.66% *v*/*v*), Gamma-T-ol (0.17, 0.27, and 0.45% *v*/*v*), Fungatol plus neem (0.12, 0.18, and 0.40% *v*/*v*), Gamma-T-ol plus neem (0.32, 0.52, and 0.85% *v*/*v*), and deionized water for control (untreated). The concentrations selected were the LC50 levels for each formulation and one concentration below and above them. Each formulation concentration and the control were replicated four times.

Spraying of mummies was done using a hand-held sprayer with the nozzle adjusted to produce a fine mist. Leaf surfaces with mummies, identified by tagging, were sprayed for run-off with 50 mL of each concentration or deionized water. After spray application, the treated plants were air-dried for 2 h and individually placed in a mesh cage (45 cm × 45 cm × 45 cm). Adult emergence from treated mummies was recorded after 7–9 days. Sprayed leaves were removed from the plants and mummies were checked under a stereomicroscope for a circular hole in the mummy, cut by an emerging adult. Mummies that did not produce wasps after seven to nine days were considered dead. Emergence for each concentration was compared with the control to determine the effects of formulations.

### 2.5. Residual Toxicity of the Tested Formulations to Adults of A. colemani under Laboratory Conditions

The adult parasitoid is the most susceptible life stage [48]. To test the susceptibility of this stage, a residual toxicity test was undertaken. The concentration selected for each formulation was one that could kill approximately 90% of a cotton aphid population (LC90), estimated in earlier toxicity tests [9]. The concentrations tested were 0.66 (Fungatol), 0.45 (Gamma-T-ol), 1.30 (Fungatol plus neem), and 1.74% *v*/*v* (Gamma-T-ol plus neem).

The test method used in this study was adopted from Grutzmacher et al. [49], with alterations. The toxicity test was undertaken by exposing adult parasitoids to fresh, dry biopesticide film sprayed on glass plates of size 12.7 × 12.7 cm, placed on the top and bottom of an exposure cage. The exposure cage consisted of an aluminum frame measuring 12.5 cm long, 1.9 cm high, and 2 cm wide. Three of the four sides of the frame had six ventilation holes (1 cm diameter), covered with transparent mesh. The fourth side had four holes: two ventilation holes (1 cm diameter) and two large holes (3 cm diameter). One of these large holes was closed with a cork and used to introduce adult parasitoids. The second was fitted with a feeding glass tube containing a cotton wick to supply sucrose solution to the adult parasitoids.

Glass plates were sprayed with 25 mL of each formulation until run-off occurred, while plates for the control (untreated) were sprayed with deionized water only. Sprayed glass plates were left to dry at room temperature, and, as soon as the spray film dried, two plates were placed over an exposure cage (one at the top and bottom). A total of 20 newly hatched mixed adult parasitoids (<24 h) were transferred into each cage. Each treatment was replicated four times. Therefore, a total of 80 adults were tested for each treatment. Mortality was recorded at 48 h after parasitoids were exposed to treatments, and longevity of the parasitoids was also recorded by checking mortality daily until all parasitoids were dead.

### 2.6. Statistical Analysis

Adult emergence, residual toxicity, and longevity data of *A. colemani* were tested for normality and homogeneity using Kolmogorov–Smirnov and Levene’s tests, respectively, by the PROC UNIVARIATE and PROC GLM procedures in SAS 9.4 [50]. Because most of our adult emergence, residual toxicity, and longevity data were normally distributed, we analyzed them using one-way ANOVA followed by Duncan’s test (PROC GLM in SAS 9.4). Due to the non-normal distribution of some data, they were compared between treatments using a non-parametric Kruskal–Wallis test (*p* < 0.05) (PROC NPAR1WAY in SAS 9.4) followed by Dunn’s pairwise comparisons.

Finally, the mortality results were used to classify the pesticide according to the four toxicity categories proposed by the IOBC for laboratory tests: Class 1, harmless (<30% reduction); Class 2, slightly harmful (30–79% reduction); Class 3, moderately harmful (80–99% reduction); and Class 4, harmful (>99% reduction) [51,52].

## 3. Results

### 3.1. Effect of the Tested Formulations on the Emergence of A. colemani from Mummies under Laboratory Conditions

**Figure 1:**
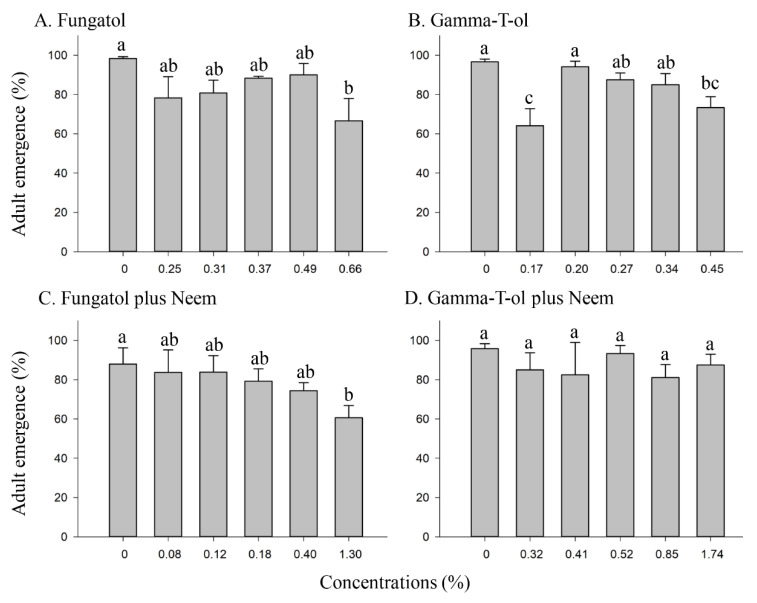
Effect of different concentrations of Fungatol (**A**), Gamma-T-ol (**B**), Fungatol plus neem (**C**), and Gamma-T-ol plus neem (**D**) on adult emergence of *A. colemani* from treated mummies in the laboratory. One-way ANOVA with the Duncan multiple comparisons test revealed significant differences between the concentrations of Fungatol (**A**), Gamma-T-ol (**B**), Fungatol with neem (**C**), and Gamma-T-ol plus neem (**D**). Different lowercase letters above bars represent significant differences at *p* < 0.05. Data presented are mean ± SEM.

Adult emergence of *A. colemani* for all concentrations of Fungatol and Gamma-T-ol was above 60%, and the highest emergence (above 96%) was observed for the control (untreated) (Figure 1A,B). Between the highest concentration of Fungatol (0.66%) and the control, significant adult emergence was detected (Figure 1A). Emergence was not concentration-related but significant differences in emergence were observed between the concentrations of Gamma-T-ol and control (F = 5.86; df = 5, 18; *p* = 0.0022) (Figure 1B). Adult emergence was 64.12 and 73.33% at the lowest and highest Gamma-T-ol concentrations (0.17 and 0.45% *v*/*v*, respectively) (Figure 1B).

When Fungatol plus neem was evaluated at various concentrations on adult emergence from the mummies, there was no significant effect (F = 1.59; df = 5, 18; *p* = 0.2129) (Figure 1C). In contrast, there was a statistically significant difference in adult emergence between the highest concentration of Fungatol plus neem (1.30%) and the control group (Figure 1C). On the other hand, there were no significant differences recorded between the Gamma-T-ol plus neem concentrations and the control (F = 0.48; df = 5, 18; *p* = 0.7884) (Figure 1D). The highest adult emergences were 84 and 93.3% at 0.12 and 0.52% concentrations for Fungatol plus neem and Gamma-T-ol plus neem, respectively (Figure 1C,D).

According to the IOBC laboratory scale, our results indicate that, in some cases, the highest tested concentrations for each formulation were slightly harmful (Class 2). However, the lowest concentrations were harmless (Class 1).

### 3.2. Effect of the Tested Formulations on the Emergence of A. colemani from Treated Mummies under Glasshouse Conditions

**Figure 2:**
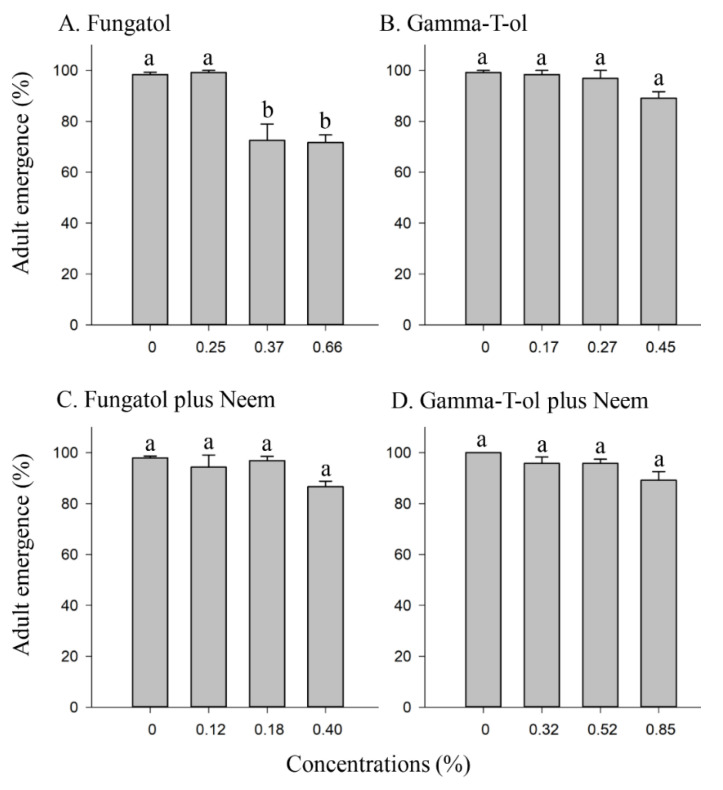
The effect of different concentrations of Fungatol (**A**), Gamma-T-ol (**B**), Fungatol plus neem (**C**), and Gamma-T-ol plus neem (**D**) on adult emergence of *A. colemani* from treated mummies in the glasshouse. The mean differences between the concentrations of Gamma-T-ol (**B**) and Gamma-T-ol plus neem (**D**) were compared using the Kruskal–Wallis (*p* < 0.05) test followed by Dunn’s pairwise comparisons. One-way ANOVA with the Duncan multiple comparisons test revealed significant differences between the concentrations of Fungatol (**A**) and Fungatol plus neem (**C**). Different lowercase letters above bars represent significant differences at *p* < 0.05. Data presented are mean ± SEM.

There was a statistically significant difference in adult emergence of *A. colemani* between the Fungatol concentrations that were evaluated and the control level (F = 18.26; df = 3, 12; *p* < 0.0001) (Figure 2A). The highest emergence (99%) of *A. colemani* was observed for the lowest concentration of Fungatol (0.25% *v*/*v*) (Figure 2A). Additionally, no significant difference in adult emergence was observed when Gamma-T-ol concentrations were compared to the control (Kruskal–Wallis chi-squared = 7.98; df = 3; *p* = 0.05) (Figure 2B). In comparison to the control, the highest concentration of Gamma-T-ol (0.45%) had the lowest emergence (89.1%) (Figure 2B). Concentrations of Fungatol plus neem had no significant effect on the emergence of *A. colemani* adults from treated mummies (F = 3.36; df = 3, 12; *p* = 0.0553) (Figure 2C). The control recorded 98% emergence, with 87% observed for the highest concentration (0.40%) of Fungatol plus neem (Figure 2C). The control for Gamma-T-ol plus neem had 100% emergence, which was not significantly different from the highest concentrations of Gamma-T-ol plus neem (Kruskal–Wallis chi-squared = 7.54; df = 3; *p* = 0.06) (Figure 2D).

### 3.3. Residual Toxicity of the Tested Formulations to Adults of A. colemani under Laboratory Conditions

**Figure 3:**
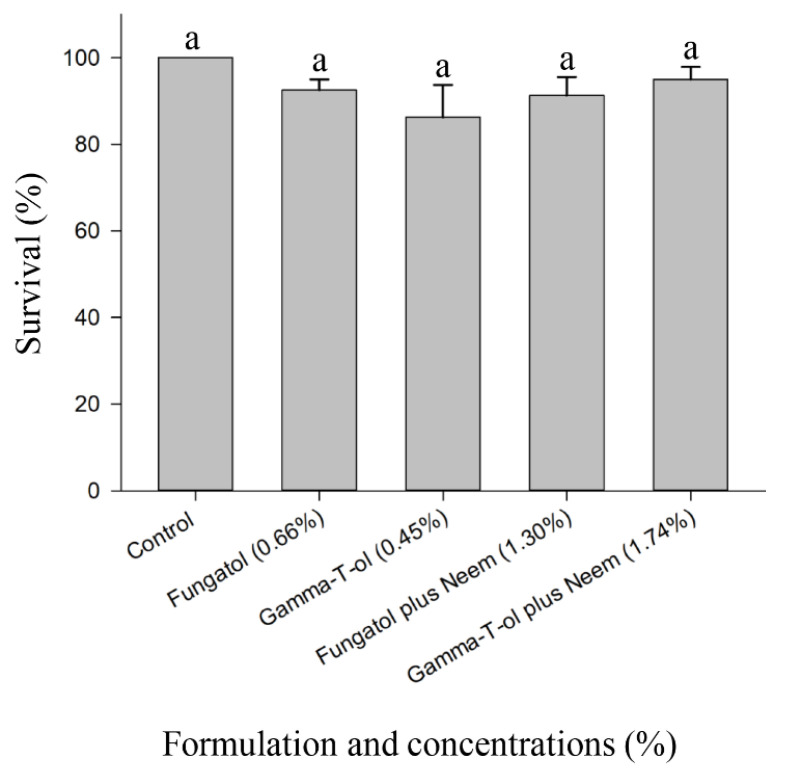
Residual toxicity of Fungatol, Gamma-T-ol, Fungatol plus neem, and Gamma-T-ol plus neem against adults of *A. colemani* in the laboratory. Each formulation was applied at a concentration resulting in 90% mortality of cotton aphids. The mean differences between the treatments were compared using one-way ANOVA test followed by the Duncan pairwise comparisons test. Different lowercase letters above bars represent significant differences at *p* < 0.05. Data presented are mean ± SEM.

Survival of adult *A. colemani* exposed to high concentrations of each formulation was between 83.8 and 97.5%, and 100% was achieved for the control (Figure 3). There was no statistically significant difference in the survival of *A. colemani* between the treatments (F = 1.44; df = 4, 15; *p* = 0.2698) (Figure 3).

**Figure 4:**
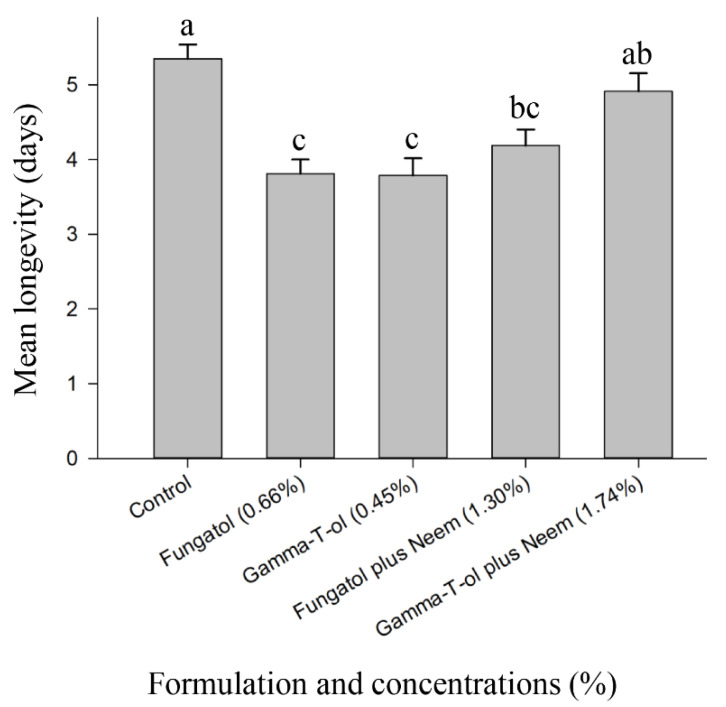
Effect of concentrations of Fungatol, Gamma-T-ol, Fungatol plus neem, and Gamma-T-ol plus neem on adult *A. colemani* longevity when exposed to formulation residues on glass plates in the laboratory. Each formulation was applied at a concentration resulting in 90% mortality of cotton aphids. One-way ANOVA with the Duncan multiple comparisons test revealed significant differences between the treatments. Different lowercase letters above bars represent significant differences at *p* < 0.05. Data presented are mean ± SEM.

The mean longevity of adult *A. colemani* exposed to residues of the formulations and control ranged from 3.74 to 5.35 days (Figure 4). There were significant differences observed between the control and the tested formulations (F = 9.47; df = 4, 95; *p* < 0.0001) (Figure 4). The lowest mean longevity was recorded at 3.74 and 3.81 days for Gamma-T-ol and Fungatol, respectively (Figure 4).

## 4. Discussion

Overall, the results of laboratory and glasshouse tests on mummies indicated that Fungatol, Gamma-T-ol, Fungatol plus neem, and Gamma-T-ol plus neem are safe to use on the parasitoid if mummification has occurred before exposure to the formulations. This was indicated by the high rates (>60%) of emergence observed for all formulations. The high emergence rates of *A. colemani* could be due to the protection of the immature parasitoid by the mummy case, as previously suggested by Borgemeister et al. [46]. As a consequence, the observations of this study agree with previous studies that identified the mummy stage as the most protected parasitoid stage [49,53]. However, according to Longley and Jepson [53], the degree of protection provided by the mummy case is dependent on factors such as the type of insecticide, dose applied, application method, and the age of the mummy. Therefore, the low toxicity observed in these trials could be due to either the nonpenetration of formulations through the mummy case or because the wasp was fully developed at the time of spray application. According to Longley [54], depending on the physio-chemical properties of the chemical, which determine its penetration and the age of the parasitoid within the mummy at the time of spraying, the parasitoid can die during its development. The stages at which this can occur are, first, at the late larval, prepupal, and pupal stages and, second, when young adults lack fully developed wings.

Fungatol and Gamma-T-ol were formulated using a variety of terpenes. In prior research, several terpenes were found to have biorational pesticidal efficacy against a variety of pest insects. For example, alpha-terpineol, alpha-terpinene, terpinen-4-ol, terpinolene, gamma-terpinene, and 1,8-cineole showed fumigation toxicity against *Drosophila melanogaster* (Meig.) (Diptera: Drosophilidae), *Reticulitermes chinensis* (Snyder) (Isoptera: Rhinotermitidae), and *Musca domestica* (L.) (Diptera: Muscidae), respectively [55,56,57]. Gaire et al. [58] discovered that terpinen-4-ol has contact and fumigation toxicity against *Cimex lectularius* (L.) (Cimicidae: Hemiptera). According to Perumalsamy et al. [59], alpha-terpineol, terpinen-4-ol, terpinolene, gamma-terpinene, and 1,8-cineole exhibited larvicidal activity against *Culex pipiens pallens* Coquillett, *Aedes aegypti* (L.), and *Ochlerotatus togoi* Theobald (Diptera: Culicidae). However, in our investigation, terpene-rich formulations were less harmful to *A. colemani*.

The adult parasitoid has been identified as the most susceptible stage [46]. The lethal and sublethal effects of the formulations on adults were assessed in terms of mortality and longevity. In the longevity test, the highest concentration of each formulation was chosen because it was slightly toxic to *A. colemani*. Fungatol, Gamma-T-ol, and Fungatol plus neem all resulted in a significant reduction in adult lifespan when compared to the control. However, in some circumstances, the combined effects of the selected formulations and neem oil were less active than the individual effects of each formulation. The reason for this could be that one molecule alters the behavior of another by altering the rate of absorption, distribution, metabolism, or excretion, as described as a possible mechanism [60]. A number of EOs have been shown to have a similar effect on lifespan, including *Azadirachta indica* (Meliaceae) seed oil, which reduces the lifespan of *Uscana lariophaga* females (Steffan) (Hymenoptera: Trichogrammatidae), *Dinarmus basalis* (Rondani) (Hymenoptera: Pteromalidae) [61], *Trichogramma pretiosum* (Riley) (Hymenoptera: Trichogrammatidae) [62], and *Trichogramma galloi* (Zucchi) (Hymenoptera: Trichogrammatidae) [63]. However, the lower concentrations of the tested formulations were not harmful to *A. colemani*. According to the IOBC laboratory scale, our results indicate that most of the lower concentrations for each formulation were harmless (Class 1). Furthermore, application of lower concentrations may not have harmful effects on the parasitoid, as it has been previously reported that application of low doses of azadirachtin (10 and 20 ppm), which is the main active component of neem oil, did not harm the hymenopteran parasitoid *Apanteles glomeratus* (Linnaeus) [64].

In comparison with previous studies, these results were similar to those that reported little or no impact of botanical pesticides on parasitoids [15,65,66,67]. At a 0.75% concentration, the EO of *Eugenia uniflora* (L.) (Myrtaceae) showed insecticidal potential on *Thaumastocoris peregrinus* (Carpintero & Dellapé) (Hemiptera: Thaumastocoridae) and was safer for the parasitoid *Cleruchoides noackae* (Lin & Hubert) (Hymenoptera: Mymaridae) [68]. When testing the effects of botanical pesticides on adults of the parasitoid *Encarsia formosa* (Gahan), Simmonds et al. [66] found that mortality when exposed to azadirachtin (4.3%) and neem (2.1%) was not significantly different from the control (0.9%). Similarly, Akol et al. [65] reported that exposure to topical sprays and residues of neem formulations had no adverse effect on the longevity and foraging behavior of adult *Diadegma mollipla* (Holmgren), a parasitoid of the diamondback moth. Mitchell et al. [69] demonstrated that neem extract negatively affected the feeding and development of *Clavigralla scutellaris* (Westwood), a coreid pest of pigeonpea, *Cajanus cajan* (L.), but did not harm its egg parasitoid, *Gryon fulviventre* (Hymenoptera: Scelionidae). Gogi et al. [70] reported that neem oil was harmless to *E. formosa* (<30% mortality). In a study by Mostafiz et al. [29], the authors discovered that methyl benzoate, a volatile organic molecule, is less toxic to the predatory bug *Nesidiocoris tenuis* (Reuter) (Hemiptera: Miridae).

In summary, tests carried out in the current study using the formulations at two life stages (larval/mummified and adult) of *A. colemani* provided important information in two areas. Firstly, treatment of mummies in the laboratory and glasshouse may assist with estimates of the survival chances of parasitoid developmental stages protected within the mummy case. Secondly, the worst-case test on the residual toxicity of formulations on adult parasitoids provided information on the chances of survival of adults, whether newly emerged or those invading treated crop canopies.

## 5. Conclusions

The results of this study indicated that Fungatol, Gamma-T-ol, Fungatol plus neem, and Gamma-Tol plus neem had low adverse effects on adult emergence from treated aphid mummies under laboratory and glasshouse conditions, as emergence for all formulations was above 60%. The results of the tests on adult survival and longevity suggest that these formulations are harmless. Based on these results, it may be proposed that the tested formulations evaluated in this study are possible botanical pesticide candidates for incorporation into an IPM program for slippery kabis. However, further trials are required to confirm these results in the field, and research should be carried out on other pests of slippery kabis and their natural enemies.

## Figures and Tables

**Table 1:** Active constituents of Alpha Tops and Gamma Tops used in this study.

	Name	Molecular Formula	Concentrations (%)
Active constituents of Alpha Tops	Terpinen-4-ol	C_10_H_18_O	60.5
	Cineole/Eucalyptol	C_10_H_18_O	22.5
	Alpha-terpineol	C_10_H_18_O	15.0
Active constituents of Gamma Tops	Gamma-terpinene	C_10_H_16_	45.5
	Terpinolene	C_10_H_16_	25.0
	Alpha-terpinene	C_10_H_16_	20.0

**Table 2:** Concentrations of each tested formulation used in mummy spraying bioassays.

Formulations/Concentrations (%, *v*/*v*)			
Fungatol	Gamma-T-ol	Fungatol Plus Neem	Gamma-T-ol Plus Neem
0.25 *	0.17	0.08	0.32
0.31	0.20	0.12	0.41
0.37	0.27	0.18	0.52
0.49	0.34	0.40	0.85
0.66	0.45	1.30	1.74

The tested concentrations for each formulation killed 30–90% of the *A. gossypii* population in our previous study [9]. * The range of the tested concentrations: LC30, 40, 50, 70, 90.

## Data Availability

There is no supplementary information to provide; all the information is contained in this manuscript.
